# Femoral nerve palsy as a complication due to COVID-19 coagulopathy and iliopsoas muscle hematoma – case report

**DOI:** 10.1186/s12891-023-07062-w

**Published:** 2023-12-06

**Authors:** Sławomir Tutak, Paweł Bartosz, Bartosz Burda, Paweł Sztwiertnia, Jerzy Białecki

**Affiliations:** 1 Centre of Postgraduate Medical Education, Orthopedic Department in Otwock, Konarskiego 13, Otwock, 05-400 Poland; 2 Radiological Department in Otwock, Gruca Orthopaedic and Trauma Teaching Hospital, Otwock, Poland

**Keywords:** COVID-19, Coagulopathy, Hematoma, Nerve, Paresis

## Abstract

**Background:**

COVID-19 (Coronavirus disease 2019) pandemic is the main medical problem around the world from the end of 2019. We found until now many symptoms of this disease, but one of the most problematic was thrombosis. Wide recommendation on COVID-19 treatment was pharmacological thromboprophylaxis. In some papers we found that clinicians face the problem of bleeding in those patients. Is still unknown that coronavirus could led to the coagulopathy.

**Case presentation:**

We described case report of patient who with COVID-19 disease present femoral nerve palsy caused by the iliopsoas hematoma. There were no deviations in coaguology parameters, patient got standard thromboprophylaxis, besides above probably COVID-19 was risk factor of hematoma formation. Non-operative treatment was applied, thrombophylaxis was discontinued. In the follow up in the radiological exam we saw reduction of the haematoma and patient report decrease of symptoms.

**Conclusions:**

We should assess individually patient with COVID-19 according to thrombosis risk factors. Probably we should be more careful in ordering thrombophylaxis medications in COVID-19 patients.

## Introduction

From the end of 2019 the world is focused on the COVID-19 (Coronavirus disease 2019) pandemic. The mortality associated with this disease and the lack of targeted treatment forces researchers into looking for symptoms and options of treatment. The virus mainly affects the respiratory tract and the main symptom are dyspnea and respiratory disorders [1]. During the pandemic, other possible symptoms caused by COVID-19 infection were also shown like: olfactory disorders, headaches, venous and arterial thrombosis, diarrhea, etc. [2]. This proves the affinity of the virus to various tissues, which may cause various symptoms.

Disorders of the coagulation system, manifested by both vascular embolism and an increased risk of bleeding, are becoming more and more important [3]. The mechanism of the coagulation disorders in COVID-19 has not been fully elucidated, but it is believed that the main cause is dysregulation of the immune system, resulting in a pro-inflammatory cytokine response, lymphocytes, hypoxia and vascular endothelial damage [4]. In those terms there were recommendations to use low-molecular heparin (LMWH) in thromboprophylaxis, which increase risk of bleeding. Despite the significant number of reports of increased blood clotting during COVID-19, there are isolated reports of an increased risk of bleeding.

The described cases of a patient with hematoma in the iliopsoas muscle during COVID-19 complicated with paresis of the femoral nerve after hip arthroplasty is an example of increased bleeding in patients with COVID-19. This case report also show potential background to further modifications of recommendation in pharmacological thrombophylaxis, specially to operated patients. We have got Institutional Bioethical Committee approval at 14.12.2022 with decision number 178/2022.

## Case description

A patient in the 70s burdened with obesity, diabetes and arterial hypertension, on 29 May 2019 she underwent left hip arthroplasty, due to hip osteoarthritis. The postoperative course was uneventful, the patient was monitored twice in the outpatient clinic, 6 weeks after the operation and 6 months after the operation. The patient had no family or genetic risk factors for the coagulation system.

**Fig. 1:**
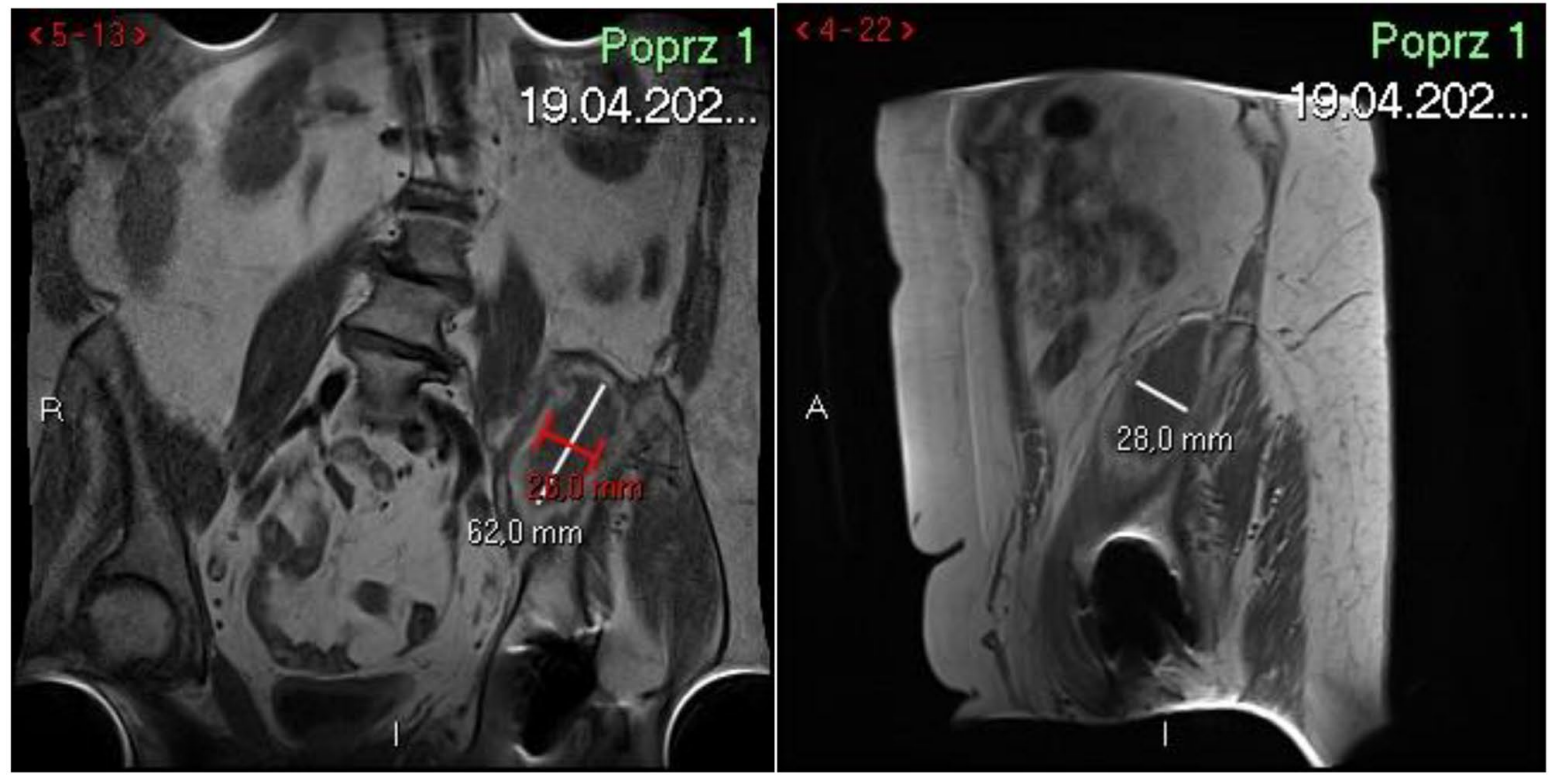
MRI scan with hematoma on the left iliopsoas muscle

In March 2021, the patient began to report shortness of breath and cough. After conducting virological diagnostics (PCR), she was diagnosed with COVID-19 infection. Due to increasing dyspnea and respiratory disorders, she was hospitalized in one of the reference hospitals. Treatment was implemented in accordance with the applicable guidelines, i.e. a prophylactic dose of anticoagulants (nodraparinum 0.6 ml 1 × 1 s.c. for 31 days), oxygen therapy and steroid (dexametashone) at a dose of 6 mg daily for 10 days. During her stay, she began to complain of pain in the left hip and in the lumbosacral region, despite of the lack of direct trauma in this area. Clinical examination revealed no active extension of the right knee joint and numbness of the right thigh; the patient was diagnosed with paresis of the left femoral nerve. Intended for outpatient treatment, where, after neurological consultation and imaging tests (USG, CT, MRI), a hematoma (127 × 79 × 51 mm) was diagnosed in the pelvis (Fig. 1). Direct pressure from the hematoma on the femoral nerve was found. In imaging exams the nerve was swollen, indicating its damage.

In April 2021, the patient was admitted to the Centre of Postgraduate Medical Education, Gruca Teaching Hospital in Otwock in order to look for the diagnosis. On admission, all laboratory parameters [morphology, CRP, renal and hepatic parameters as well as parameters of the coagulation (PT 13.8 s, APTT 28.1 s, INR 1.04)] were normal. Imaging tests (abdominal CT, abdominal ultrasound and MRI of the abdominal cavity and pelvis did not report a kidney problem. The only aspect of the urinary system was episode of infection during last hospitalization. This was confirmed in urine culture test and treated with direct antibiotic therapy. The patient discontinued to receive a prophylactic dose of anticoagulants. After re-neurological consultation, weakness of the left hip joint flexion and lack of active extension of the left knee joint, decreased tension of the quadriceps muscle of the thigh (1 in the Lovette scale), decreased superficial sensation on the anterior and medial surface of the thigh and the medial surface of the shin were found. Due to the lack of a suitable device, it was not possible to perform an EMG examination during hospitalization. Further imaging exams showed decreasing of the hematoma size (approx. 62 × 26 × 28 mm).

The patient was qualified for conservative treatment. Physical therapy and rehabilitation were implemented. The patient underwent control Doppler ultrasound examinations in order to exclude venous thrombosis of the lower limbs, no thrombotic changes were found in any of the exams. Low molecular weight heparins (LMWH) were withdrawn due to the prevailing risk of bleeding.

**Fig. 2:**
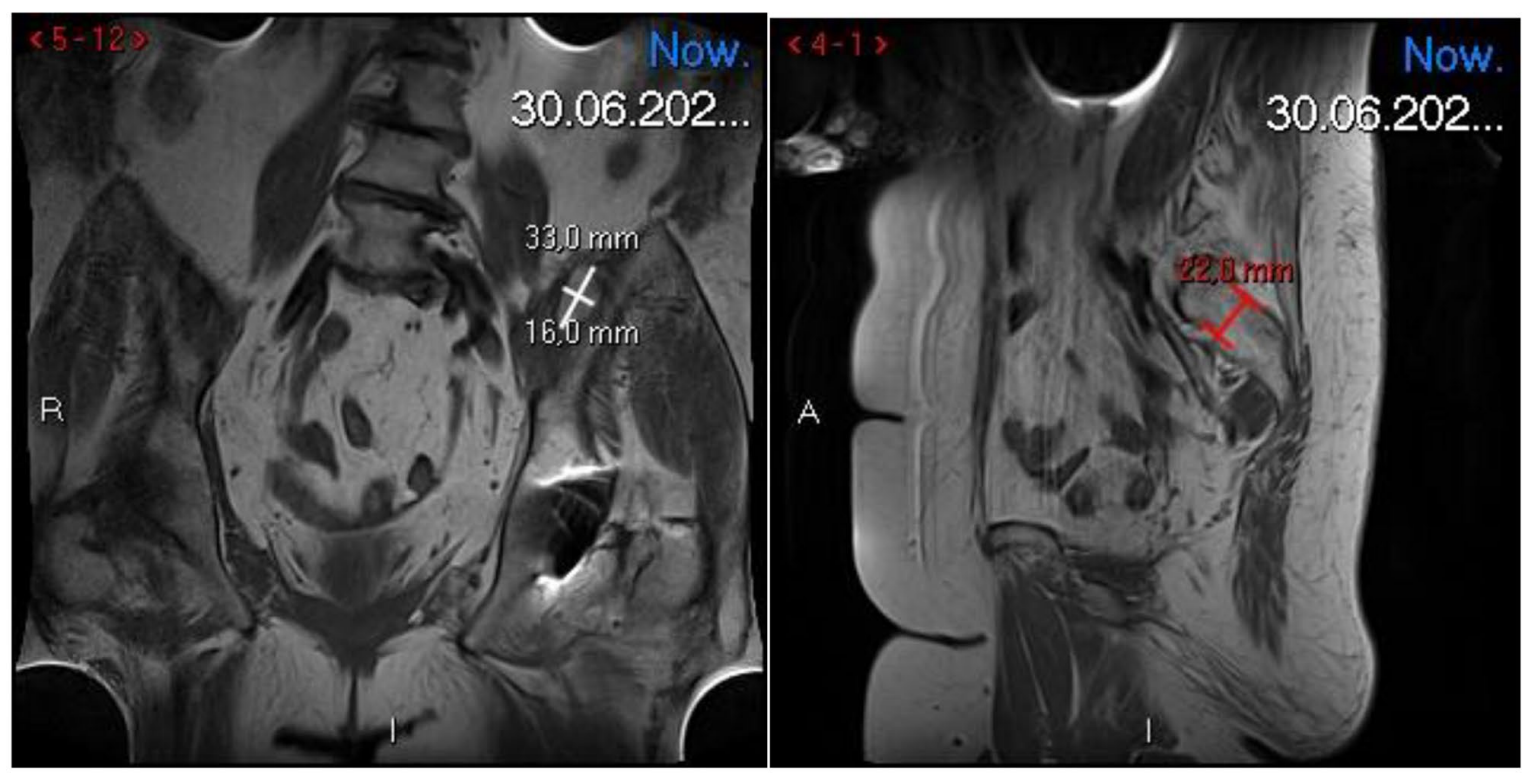
Control MRI with regression of hematoma

After another two months, the patient was re-admitted to the Department. The laboratory parameters were normal again, and the coagulation tests did not change significantly (PT 13.7s, APTT 30.0s, INR 1.04). Follow-up MRI scans were performed. MRI showed a reduction in the size of the hematoma to 33 × 16 × 22 mm [Fig. 2], while ultrasound showed the left femoral nerve with a preserved continuity, normal echostructure, non- thickening, and no signs of focal neuropathy.

On neurological examination, the patient showed slight improvement (2 in the Lovette scale), but the symptoms of femoral nerve paresis were still present.

After the hospitalization, the patient was ordered regular (3 times a week) physiotherapy. After rehabilitation, the symptoms of femoral nerve palsy decreased significantly. The active flexion movement in the hip joint (slightly weakened) and extension in the knee joint returned (4 in the Lovette scale).

The area of cutaneous hypoaesthesia on the inside of the thigh and lower leg also decreased. This improvement allowed the patient to replace the high walker (which she had been using until December 2021) for elbow crutches. The subjective quality of life improved significantly.

**Fig. 3:**
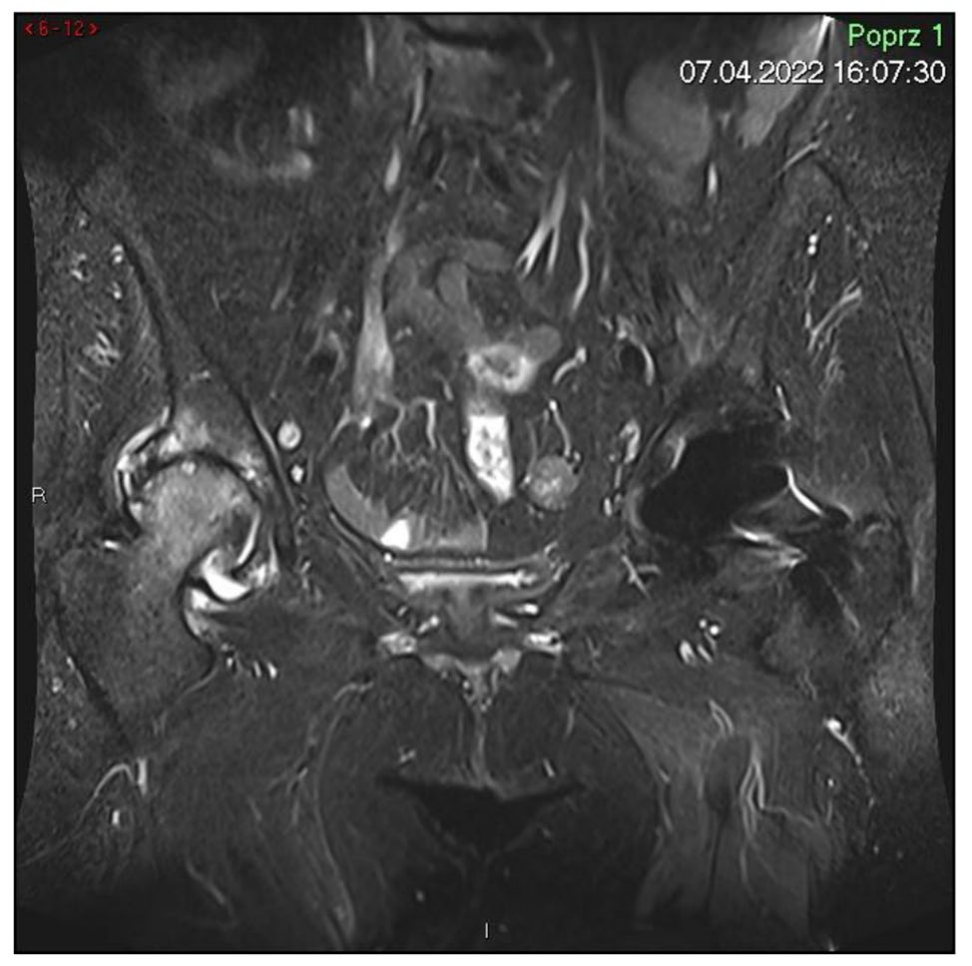
Last MRI scan with full hematoma regression

On April 7, 2022 a control MRI examination was performed [Fig. 3]. Compared to the previous study, the hematoma in the left hip muscle was absorbed, and in this localization there are no visible fibrosis and a strip of edema. Additionally, intensification of degenerative changes in the right hip joint was found.

At the last visit to the outpatient clinic in June 2022, the patient no longer reports problems with the left lower limb. The limb function has fully recovered, but she still has used crutches because of right hip. The patient was qualified to total hip replacement.

## Discussion

The published case of a patient with palsy of the femoral nerve as a result of a hematoma in the iliopsoas muscle area sheds light on another therapeutic problem related to COVID-19. Although it is still a poorly understood disease, many publications mention the association COVID-19 with increased blood clotting [5, 6]. Al-Samkari estimated the percentage of bleeding complications at 4.8% [3].

The problem of increased bleeding in patients with COVID-19 is not fully understood. In publications on this subject, one of the listed reasons for increased bleeding is the use of LMWH in treatment. According to the recommendations of the American Society of Hematology, patients hospitalized due to COVID-19 require antithrombotic prophylaxis with prophylactic doses of low molecular weight heparins [7]. The use of therapeutic doses of LMWH in patients with severe disease is increasingly recommended. The above measures increase the risk of bleeding [8]. According to Ishan Paranjpe, among patients receiving therapeutic doses of LMWH, 63% of them had an episode of so-called “major bleeding” [9]. The Musoke study shows a high death rate due to the so-called “major bleeding” associated with therapeutic doses of LMWH, with particular emphasis on the 100% mortality due to central nervous system bleeding. According to Musoke, the decision to include LMWH in patients with COVID-19 should be based on standards of care, with particular emphasis on the indications for the use of LMWH in therapeutic doses, i.e. VTE and pulmonary embolism [10].

According to the literature, another factor contributing to bleeding complications directly related to SARS-CoV 2 infection is autoimmune thrombocytopenia (ITP). According to Guan, the idiopathic thrombocytopenic purpura percentage in COVID-19 is 36%. It is usually mild [11]. The causes of ITP include some viral and bacterial infections, medications, and recently also vaccinations [12, 13].

In the described case, only prophylactic doses of LMWH were used in the patient. Laboratory tests showed no abnormalities. In the absence of other visible causes of the increased risk of bleeding complications, the cause of hematoma in the iliopsoas muscle area and secondary femoral nerve palsy should be associated with COVID-19 disease and LMWH intake.

This article has some limitations. The main one is that we have not done serological or genetical tests into congenital coagulopathy. There were no patients history in such diseases. Maybe future studies should consider such tests to find correlation between COVID-19 disease and such conditions.

The above case is a field for further discussion about the possible side effects of anticoagulants and the need to evaluate the potential gains and losses in the use of LMWH prophylaxis in this disease. While the doctors treating severe cases of COVID-19 with non-obvious veinthrombosis symptoms is still main indication to implement antithrombotic treatment. The discussed case argues for the need to conduct further research on the SARS-CoV-2 virus and its relationship to bleeding complications.

## Data Availability

The datasets used and analysed during the current study are available from the corresponding author on reasonable request.

## Abbreviations

COVID-19: Coronavirus disease 2019
LMWH: Low molecular weight heparin
PCR: Polymerase chain reaction
CT: Computer tomography
MRI: Magnetic resonance imaging
USG: Ultrasonography
CRP: C–reactive protein
PT: Prothrombin time
APTT: Activated partial thromboplastin clotting time
INR: International normalized ratio
ITP: Idiopathic thrombocytopenia
SARS Cov-2: Severe acute respiratory syndrome coronavirus 2
